# Federated Deep Reinforcement Learning Based Task Offloading with Power Control in Vehicular Edge Computing

**DOI:** 10.3390/s22249595

**Published:** 2022-12-07

**Authors:** Sungwon Moon, Yujin Lim

**Affiliations:** Department of IT Engineering, Sookmyung Women’s University, Seoul 04310, Republic of Korea

**Keywords:** vehicular edge computing, task offloading, power control, deep deterministic policy gradient, federated deep reinforcement learning

## Abstract

Vehicular edge computing (VEC) is a promising technology for supporting computation-intensive vehicular applications with low latency at the network edges. Vehicles offload their tasks to VEC servers (VECSs) to improve the quality of service (QoS) of the applications. However, the high density of vehicles and VECSs and the mobility of vehicles increase channel interference and deteriorate the channel condition, resulting in increased power consumption and latency. Therefore, we proposed a task offloading method with the power control considering dynamic channel interference and conditions in a vehicular environment. The objective is to maximize the throughput of a VEC system under the power constraints of a vehicle. We leverage deep reinforcement learning (DRL) to achieve superior performance in complex environments and high-dimensional inputs. However, most conventional methods adopted the multi-agent DRL approach that makes decisions using only local information, which can result in poor performance, while single-agent DRL approaches require excessive data exchanges because data needs to be concentrated in an agent. To address these challenges, we adopt a federated deep reinforcement learning (FL) method that combines centralized and distributed approaches to the deep deterministic policy gradient (DDPG) framework. The experimental results demonstrated the effectiveness and performance of the proposed method in terms of the throughput and queueing delay of vehicles in dynamic vehicular networks.

## 1. Introduction

With technological advancements in communication, computing, and sensing, vehicular networks have expanded to the Internet of Vehicles (IoV) [1]. IoV facilitates vehicular applications such as intelligent navigation and crowd sensing and a large number of smart vehicles are moving on the road. Most vehicular applications are computation-intensive, and it is difficult for vehicle terminals to process these tasks due to hardware constraints and power considerations [2].

Therefore, vehicles offload their tasks to cloud and edge servers for execution. Task offloading can improve the quality of service (QoS) of computation-intensive and delay-sensitive applications. Cloud servers provide sufficient and fast computational resources, but the large amount of data delivered to the central cloud causes network congestion and unpredictable delays. Thus, vehicular edge computing (VEC), which deploys a VEC server (VECS) at the edge of the network near vehicles, is a promising solution to address these challenges [3]. Offloading tasks to the VECS can reduce network congestion, delays, and energy consumption of vehicles.

Although VEC has numerous advantages, several challenges remain. While vehicular applications are sensitive to latency requirements, service latency is affected by various offloading factors, e.g., transmission and power allocation. Therefore, the efficiency of wireless data transmission during offloading must be considered. The channel condition is dynamic and uncertain owing to path loss and channel interference caused by the mobility of a vehicle. The density of vehicles, VECSs, and mobility of vehicles increase channel interference and deteriorate the channel condition, resulting in increased power consumption and latency. It is important to optimize the offload decision, considering channel interference, conditions, and power consumption. 

With the development of deep neural networks (DNN), deep reinforcement learning (DRL) has become an advantageous framework for solving decision-making problems, especially complex problems such as resource allocation in wireless communication networks [4]. Many of the existing methods using the DRL framework consider single-agent [5,6] or multi-agent [7,8]. In single-agent methods, the central controller/base station (BS) collects global information to determine the action of each vehicle. In multi-agent methods, each vehicle collects local observations and selects its action as an agent. 

Federated learning (FL) was proposed to leverage both centralized and distributed methods [9]. FL allows each device to train a network model, collect the model parameters, and transmit them to a central server. By repeatedly sending messages or model updates on a small scale rather than transmitting the entire data, FL can reduce communication costs [10]. In addition, communication costs can be reduced through power control. Power control ensures that the transmission power of each vehicle meets the communication requirements, maximizes the throughput of the VEC system, meets the QoS requirements, and prevents unnecessary interference with other signals.

In this study, we propose task offloading with power-control-based on the FL of the DRL method in a dynamic VEC system. We focus on allocating the transmission power for offloading to maximize the throughput of the VEC system within the power constraints of the vehicle. We formulate the task offloading with the power control method as a Markov decision process (MDP) and compare the performance and efficiency of the single-agent, multi-agent, and federated versions of the method. The contributions of this study can be summarized as follows:

1. We formulate a task offloading problem with power control in a dynamic VEC system where the channel condition is dynamic and uncertain owing to path loss and channel interference. The objective is to maximize the throughput of the VEC system within the power constraints of the vehicle;
2. The optimization problem is allocating the transmission power of the vehicle, so it is considered a continuous decision-making problem. Therefore, a DRL framework based on DDPG is proposed to solve this problem. DDPG is a combination of deep Q-network (DQN) and actor–critic (AC), which can solve the decision-making problem of continuous action space;
  - FL is introduced into the DRL to improve training performance. Each vehicle trains the model with its own local information. Then, the parameters of the learned models are uploaded and aggregated into a VEC controller. Therefore, FL has advantages of both centralized and distributed methods;
  - The experimental results show that our proposed method, FL-DDPG, outperforms other comparison methods in convergence and performance in terms of throughput and queueing delay.

The remainder of this paper is organized as follows: We review the related works in Section 2. In Section 3, we introduce the system model, and the problems are formulated in this section. In Section 4, we describe an offloading method based on the FL of the DRL. In Section 5, the performance of the methods is analyzed using experimental results. Finally, in Section 6, we conclude the paper.

## 2. Related Work

In this section, we review the related works on task offloading with resource allocation based on DRL. There are several offloading methods based on single-agent and multi-agent DRLs. In single-agent methods, it becomes difficult to make an optimal decision as the size of the VEC system increases in terms of the number of vehicles and VECSs. Moreover, this causes huge overhead, extra latency, and privacy issues for vehicles. 

Therefore, many recent works have been proposed based on multi-agent DRL, which can efficiently reduce communication overhead and latency, and vehicles train and execute the model independently without sharing information with others. In [11,12], methods based on DRL, namely DQN, which combines DNN with Q-learning, have been proposed. However, these methods are based on a discrete action space. Therefore, there is a limit to dealing with continuous values, such as power. 

A DRL named deep deterministic policy gradient (DDPG), based on a continuous action space, was applied to the offloading method. In [13], task offloading with a power control method based on DDPG was proposed to maximize the long-term system utility, including the total execution latency and energy consumption. In [14], DDPG-based task offloading and the power allocation method were proposed to minimize long-term energy consumption while satisfying the latency constraints of mobile devices. In [15], the authors proposed a DDPG-based method to optimize an offloading policy that minimizes the total latency cost and energy consumption. However, these existing methods focus on the performance improvement in a quasi-static environment without considering a dynamic VEC system. Since they did not consider uncertain channel conditions in the MDP formulation, they optimized offloading or power allocation using predefined criteria. To solve the limitation, we reformulate the problem without predefined criteria. In other words, we consider dynamic channel condition, interference, and mobility of vehicle in the MDP formulation.

Some studies have proposed an offloading method in a dynamic environment. In [16], offloading with a resource allocation method based on DDPG was to optimize the allocation of power and local execution resources under a dynamic environment consisting of mobile devices. The goal is to minimize the long-term cost, which consists of offloading delay and energy consumption for mobile devices. In [17,18], offloading with power allocation based on DDPG methods was proposed to optimize the power allocation for transmission and local power. The reward function was modeled based on task buffer size and power consumption. Many existing studies have considered both offloading and local execution. However, the vehicle has limited energy and resources, and it takes a lot of energy to train learning models, make decisions, execute tasks, and offload. In this study, we consider only offloading decision compared to existing methods. Therefore, we proposed a method based on the DDPG framework to determine the amount of task to be offloaded considering the power consumption of the vehicle.

We leveraged the FL of the DRL framework to take advantage of both centralized and distributed networks. However, thus far, FL has been applied to supervised learning problems in fields such as machine vision and natural language processing (NLP). There have been fewer studies that use the FL to train DRL models and distributed control [10]. In [19], task offloading and resource allocation methods based on FL-DDPG were proposed to minimize the energy consumption of devices under latency constraints and limited resources. In [20], the authors proposed offloading methods based on an FL-DDQN to reduce the transmission costs between devices and edge servers. The FL agent, which acts as a server for the FL process, is working on edge server. However, the vehicular environment is affected by the limited coverage of VECSs, which causes several problems during the offloading procedure. In addition, FL requires a central server that can collect information from all the vehicles. For VECSs acting as FL agents, the limited coverage of VECS reduces the number of vehicles that can participate in the training process. The FL process convergence is significantly influenced by the number of FL devices participating in it [21]. In this study, we propose an offloading method by adopting the DDPG-based FL and consider a VEC controller as an FL agent that can operate from a more global perspective while implementing FL in a vehicular environment. 

## 3. System Model and Problem Formulation

**Figure 1:**
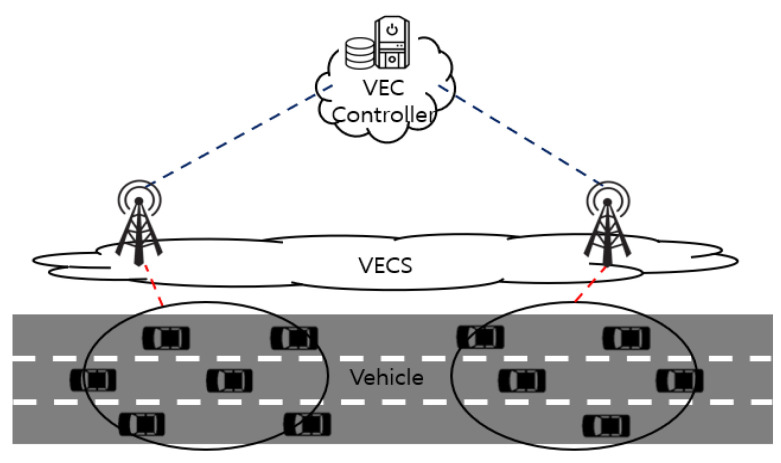
System model.

As depicted in Figure 1, the system model consists of three layers: the vehicle, VECS, and VECS controller. The tasks should be offloaded to the VECS for processing because the vehicle has limited computing resources and energy. The VECS has a powerful computing capacity for processing tasks offloaded by vehicles. The VECS controller plays an auxiliary role, which aggregates the neural network parameters of each VECS to help the VECS to take better decisions.

Let us consider a VEC system in which a set of VECSs, M, is placed on the road, and a VECS is attached to a roadside unit (RSU). The set of vehicles, N, is moving on the road at a speed $v_{n},n\in N$ within the coverage of the RSU. The time is divided into slots with a duration $\tau_{0}$. At each slot, each vehicle generates tasks following an independent and identical distribution (i.i.d.) based on the mean task arrival rate $\lambda_{n}=E\left[z_{n}\left(t\right)\right]$, where $z_{n}\left(t\right)$ is a quantification of the number of tasks at time slot t. These tasks arrive at the task queue of each vehicle n, which has a limited queue operating in a first-come-first-service (FCFS) manner. Each vehicle allocates transmission power to offload tasks to the VECS to handle tasks stored in the queue. Therefore, the number of tasks that vehicle n offloads to VECS m,m∈M at time t is expressed as follows:

(1) $$d_{n,m}\left(t\right)=B_{n}log\left(1+\gamma_{n,m}\left(t\right)\right)$$

where $B_{n}$ is the bandwidth associated with vehicle n during communication, and $\gamma_{n,m}\left(t\right)$ is the signal-to-interference-plus-noise ratio (SINR) of vehicle n in VECS m at time t, which is calculated by the following equation:

(2) $$\gamma_{n,m}\left(t\right)=\frac{p_{n,m}\left(t\right)g_{n,m}\left(t\right)}{\sigma^{2}+\sum_{n^{\prime }\neq n}p_{n^{\prime },m}\left(t\right)g_{n^{\prime },m}\left(t\right)+\sum_{m^{\prime }\neq m}g_{n,m^{\prime }}\left(t\right)\sum_{n^{\prime }}p_{n^{\prime },m^{\prime }}\left(t\right)}$$

where $p_{n,m}\left(t\right)\in \left[0,\;P_{max}\right]$ is the transmission power from vehicle n to VECS m, and $P_{max}$ is the maximum transmission power constraint, respectively. $\sigma^{2}$ is the noise power, and $\sum_{n^{\prime }\neq n}p_{n^{\prime },m}\left(t\right)g_{n^{\prime },m}\left(t\right)$ and $\sum_{m^{\prime }\neq m}g_{n,m^{\prime }}\left(t\right)\sum_{n^{\prime }}p_{n^{\prime },m}\left(t\right)$ denote the intra-cell interference and inter-cell interference, respectively. Further, $g_{n,m}\left(t\right)$ is the channel gain between vehicle n and VECS m at time slot t which is calculated as follows:

(3) $$g_{n,m}\left(t\right)={\left|h_{n,m}\left(t\right)\right|}^{2}\beta_{n,m}$$

where $h_{n,m}\left(t\right)$ and $\beta_{n,m}$ denote the small-scale fading and the large-scale fading, including, reflects path loss and shadowing, respectively. The relationship between $h_{n,m}\left(t\right)$ and $h_{n,m}\left(t-1\right)$ is formulated as:

(4) $$h_{n,m}\left(t\right)=\rho_{m}h_{n,m}\left(t-1\right)+\sqrt{1-\rho_{m}^{2}}e_{n,m}\left(t\right)$$

where $e_{n,m}\left(t\right)$ is the error vector correlated with $h_{n,m}\left(t\right)$ and followed by the complex Gaussian distribution. Moreover, $\rho_{m}$ is the normalized channel correlation coefficient between the time slots t−1 and t, which is calculated as follows:

(5) $$\rho_{m}=J_{0}\left(2\pi f_{d}^{n}\tau_{0}\right)$$

where $J_{0}\left(\cdot \right)$ is the first-kind zero-order Bessel function, and $f_{d}^{n}$ is the maximum Doppler frequency of vehicle n, which is calculated by the following equation:

(6) $$f_{d}^{n}=\frac{v_{n}}{\Lambda }cos\Theta$$

where Θ is the angle between the direction of movement of the vehicle and the uplink communication direction, and Λ is the wavelength, respectively. 

The amount of task $z_{n}\left(t\right)$ is generated, and the amount of task $d_{n,m}\left(t\right)$ is offloaded to the VECS for processing, which is calculated by Equation (1). Therefore, the queue length *q_n_*(*t*) of vehicle n in slot t is expressed as follows:

(7) $$q_{n}\left(t+1\right)={\left[q_{n}\left(t\right)-d_{n,m}\left(t\right)\right]}^{+}+z_{n}\left(t\right)$$

where ${\left[\cdot \right]}^{+}=\max\left(0,\cdot \right)$.

The goal of this study is to optimize the long-term reward in terms of the throughput of the VEC system under the power constraints of tasks by dynamically allocating the transmission power for offloading as follows:

(8) $$\max\sum_{n}\sum_{m}d_{n,m}$$

(9) $$s.t.\;0\leq \;p_{n,m}\left(t\right)\leq P_{max}$$

where Equation (9) indicates that transmission power of vehicle n cannot exceed the constraint on the maximum transmission power. 

This problem is non-convex because there are interference terms in the denominator of the SINRs. To optimize the problem, the method should access the channel state information for all vehicles, but the vehicles as agents can only access partial observations of the environment. Therefore, to achieve better performance, we reformulated the problem of tuning the offloading decision based on the feedback received from the controller when each vehicle made a decision.

## 4. Proposed Method

In this section, we propose an FL for DRL-based offloading with a power control method in a dynamic VEC system. A DRL-based DDPG and FL are used to optimize the transmission power for offloading to maximize the throughput of the VEC system under the power constraints of vehicles.

### 4.1. MDP Formulation

We formulated the offloading problem as an MDP, where S is a set of states, A is a set of actions, and R is an immediate reward. Each vehicle is considered an agent, and the agent observes a state $s_{t}$, and chooses an action $a_{t}$ based on its observations of the environment at slot t. Thereafter, the agent receives the reward $r_{t}$ and transitions from the state $s_{t}$ to the next state $s_{t+1}$. We defined these three MDP components as follows:

- State: Each vehicle agent observes its state at slot t to optimize the offloading policy. The transmission power is affected by uncertain channel conditions owing to the mobility of vehicles and channel interference. Therefore, the local state of the vehicle should reflect the SINR, $\gamma_{n,m}\left(t\right)\;$of vehicle *n* in VECS m for the uncertain channel condition (Equation (2)) and the queue length, $q_{n}\left(t\right)$ of vehicle *n* for the stochastic task arrival (Equation (7)). Moreover, we considered the previous transmission power to perform better initialization because the last solution is correlated in the time domain [22]. Accordingly, the state of vehicle n is defined as follows:
  (10) $$s_{n}^{t}=\left[\gamma_{n,m}\left(t\right),q_{n}\left(t\right),p_{n,m}\left(t-1\right)\right]$$
- Action: Each vehicle agent allocates the transmission power for offloading to the VECS m based on the local state $s_{n}^{t}$. Accordingly, the action of vehicle n is defined as follows:
  (11) $$a_{n}^{t}=p_{n,m}\left(t\right)$$
- Reward: Since the reward function is associated with the objective, it is defined using the objective of the optimization problem (Equation (8)). Therefore, we defined the reward function to maximize the amount of data that the agent offloads while alleviating its interference with adjacent links. Accordingly, the reward function is defined using as follows:
  (12) $$r_{n}^{t}=d_{n,m}\left(t\right)+\omega \cdot \sum_{m^{\prime }\neq m}\sum_{n^{\prime }}d_{n^{\prime },m^{\prime }}\left(t\right)$$
  where ω is a weighted parameter between the throughput and interference of the agent.

### 4.2. DDPG Formulation

DDPG is a method that supports continuous action spaces and is based on an actor–critic framework. DDPG adopts DNNs to act for policy improvement and to criticize the policy evaluation, which is the reason for naming it the actor and critic networks, respectively. Through iterative policy improvements and evaluations, DDPG can obtain the optimal policy. The input and output of the actor network are state and determined action values, respectively. The input and output of the critic network are state and action, and *Q*-value, respectively. The actor network consists of the evaluation network μ, and the target network μ′. The critic network consists of an evaluation network Q, and target network Q′. The corresponding parameters of these four networks are $\theta^{\mu }$, $\theta^{\mu \prime }$, $\theta^{Q}$, and $\theta^{Q\prime }.$ By adopting experience replay memory to break up the correlation among training samples, experience tuples are stored in each training step. 

Each vehicle agent n aims to maximize its expected discounted long-term reward, where $Q_{\pi }\left(s_{n}^{t},a_{n}^{t}\right)$ is the action-value function under policy π as follow:

(13) $$Q_{\pi }\left(s_{n}^{t},a_{n}^{t}\right):=E_{\pi }[\sum_{t=1}^{\infty }\gamma^{t-1}r_{n}^{t}]$$

where γ is the discount factor to determine the future reward. It was proved that solving $\nabla_{\theta^{\mu }}J$ can be replaced by solving $\nabla_{\theta^{\mu }}Q_{\pi }\left(s_{n}^{t},a_{n}^{t}\right)$*,* which is the gradient of $Q_{\pi }\left(s_{n}^{t},a_{n}^{t}\right)$ [23]. However, due to the continuous action space, $Q_{\pi }\left(s_{n}^{t},a_{n}^{t}\right)$ of Equation (11) cannot be calculated by the Bellman equation. To address this problem, the critic network adopts a DNN parameterized with $\theta^{Q}$ to approximate the action-value function $Q_{\pi }\left(s_{n}^{t},a_{n}^{t}\right)$ represented by $Q\left(s_{n}^{t},a_{n}^{t}|\theta^{Q}\right).$ The critic network updates its parameters according to the loss function, as follows:

(14) $$L\left(\theta^{Q}\right)=E\left[{\left(y_{n}^{t}-Q\left(s_{n}^{t},a_{n}^{t}|\theta^{Q}\right)\right)}^{2}\right]$$

(15) $$y_{n}^{t}=r_{n}^{t}+\gamma Q(s_{n}^{t+1},\mu \prime (s_{n}^{t+1}|\theta^{\mu \prime })|\theta^{Q\prime })$$

where $Q\left(s_{n}^{t},a_{n}^{t}|\theta^{Q}\right)$ and $y_{n}^{t}$ are the *Q*-values approximated by the evaluation network and target network, respectively. In addition, $\mu \prime (s_{n}^{t+1}|\theta^{\mu \prime })$ refers to the action taken by the target actor network at $s_{t+1}$. 

The actor network updates its parameters according to the direction proposed by the critic network, and the policy gradient for this can be calculated as follows:

(16) $$\begin{array}{l} \nabla_{\theta^{\mu }}J\\ \;\approx \frac{1}{N}\underset{t}{\sum }\nabla_{\theta^{\mu }}Q\left(s_{n}^{t},a_{n}^{t}|\theta^{Q}\right){|}_{\;a_{n}^{t}=\mu \left(s_{n}^{t}\right)}\\ =\frac{1}{N}\underset{t}{\sum }\nabla_{a_{n}^{t}}Q\left(s_{n}^{t},a_{n}^{t}|\theta^{Q}\right){|}_{\;a_{n}^{t}=\mu \left(s_{n}^{t}\right)}\cdot \nabla_{\theta^{\mu }}\mu (s_{n}^{t}|\theta^{\mu }) \end{array}$$

Each agent slowly updates the parameters of the target networks of the critic and actor as follows:

(17) $$\theta^{Q\prime }\leftarrow \tau \theta^{Q}+\left(1-\tau \right)\theta^{Q\prime }$$

(18) $$\theta^{\mu \prime }\leftarrow \tau \theta^{\mu }+\left(1-\tau \right)\theta^{\mu \prime }$$

where τ is the update parameter for target networks. 

### 4.3. FL-DDPG Formulation

We propose an FL-DDPG method using FL-based DDPG. The greater the amount of data, the better the training performance in the neural network training process. However, they are reluctant to transit the raw data to the central server for data privacy and security. Thus, FL realizes joint modeling based on centralized and distributed learning, further ensuring data privacy and security, and improving the performance of the model. 

Let us consider the VEC controller as an FL agent, which collects the parameters of models from the vehicles and creates new global update parameters. Each vehicle downloads the model from the VEC controller to train it with its own observations. The training parameters are thereafter returned to the VEC controller for aggregation and updating. Through iterations between distributions and aggregations, we can obtain better training models without sharing the raw data. The aggregation of parameters can be expressed as follows:

(19) $$\Theta =\frac{1}{N}\sum_{n\epsilon N}\Theta_{n}$$

where $\Theta_{n}$ denotes the parameters of local model for vehicle n, which means the updated parameters of the target Q-network. Θ denotes the averaged parameters of the global model for the VEC controller, which means the averaged parameters of target Q-networks. The procedure for the FL-DDPG-based offloading method is presented in Algorithm 1.
**Algorithm 1** FL-DDPG-based Offloading MethodInitialize actor network μ and critic network Q Initialize target network with weights μ’ and critic network Q’ Initialize experience replay memory **for** each episode e∈E **do** Initialize parameters for simulation in VEC environment  Generate randomly an initial state $s_{n}^{t}$ for each vehicle agent n∈N **for** each time slot t∈T **do**  **for** each agent n∈N **do**   Determine transmission power for offloading by selecting an action   $a_{n}^{t}=\mu \left(s^{t}\right)+\Delta \mu$, Δμ is a sampled noise   Execute the action $a_{n}^{t},\;$receive reward $r_{n}^{t}$, and observe new state $s_{n}^{t+1}$   Store the tuple $\left(s_{n}^{t},\;a_{n}^{t},r_{n}^{t},s_{n}^{t+1}\right)$ into experience replay memory   Sample randomly a mini-batch of samples from experience replay memory   Update the critic and actor network via Equations (14) and (16)    Update target networks via Equations (17) and (18) **If** episode e == $e_{aggregation}$ **then**  Upload the parameters to the VECS controller according to Equation (19)  Download the parameters from the VECS controller to each vehicle

## 5. Simulation

In this section, we evaluate the proposed method against other methods. Simulations were implemented using Pytorch 1.4.0 with Python 3.6, and all vehicles have mobility with dataset [24] in an area of 2.5 × 1.5 km using the SUMO simulator [25]. The actor and critical networks of DDPG are composed of four fully connected neural networks with two hidden layers, with 128 and 64 neurons, respectively. The ReLU activation function was used for all hidden layers. The learning rate of the actor was $10^{-4}$ and of the critic network was $10^{-3}$. The experience replay memory size was 2.5 $\times 10^{5}$, and the batch size was 128, respectively. The remaining parameters used in the simulations are listed in Table 1. 

The proposed method, FL-DDPG, was compared with the single-agent and multi-agent versions of DDPG, S-DDPG, and M-DDPG, respectively. To evaluate the performance of the proposed method based on DDPG, which operates on a continuous action space, we compared it with DQN methods. Since DQN operates on a discrete action space, the power level for transmission is defined as $P=\{\frac{kP_{max}}{l}|k=1,\ldots ,l\}$, where the number of power levels is set as l=10. Therefore, we compared it with the single-agent, multi-agent, and FL versions of DQN and greedy methods.

- FL-DDPG: FL-DDPG is a method proposed in this study. FL is introduced into DDPG. Each vehicle trains the model, and the VEC controller aggregates the parameters of the trained models and updates it to a global model;
- S-DDPG (single-agent DDPG): As a single DDPG agent, the VEC controller collects information from all vehicles and trains the model with DPPG;
- M-DDPG (multi-agent DDPG): Each vehicle that is a DDPG agent trains the model independently with DDPG without sharing information each other;
- FL-DQN: FL is introduced into DQN. Each vehicle trains the model independently, and the VEC controller trains the model in a centralized approach;
- S-DQN (single-agent DQN): The VEC controller as a single DQN agent trains the model with DQN in a centralized approach;
- M-DQN (multi-agent DQN): Each vehicle as a DQN agent trains the model with DQN in a distributed approach;
- Greedy power (GD-P): For each slot, each vehicle agent offloads the tasks to the VECS using the maximum transmission power without considering channel interference.

**Figure 2:**
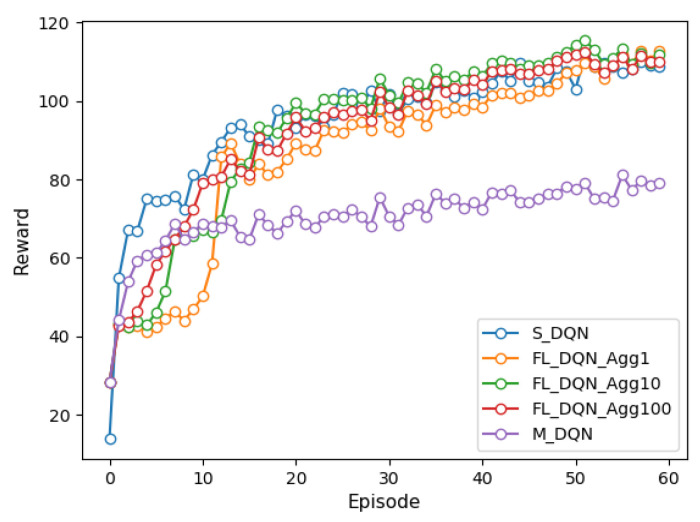
Comparison of reward of DQN methods under episodes.

**Figure 3:**
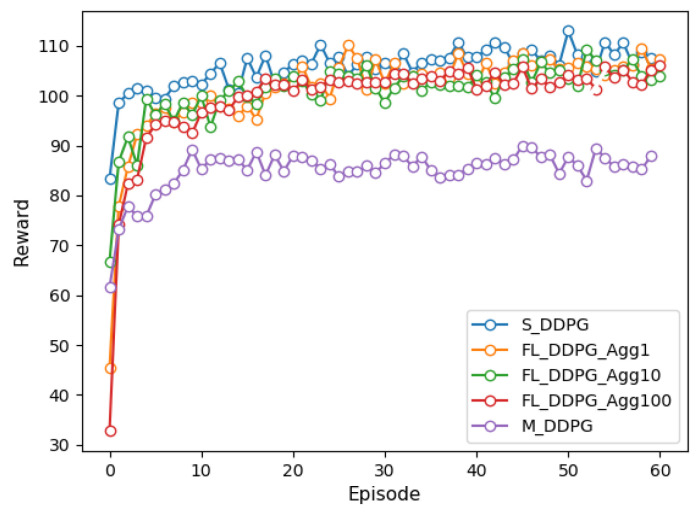
Comparison of reward of DDPG methods under episodes.

Figure 2 and Figure 3 depict the reward for single-agent, multi-agent, and federated versions of the DQN and DDPG methods under episodes. We trained each model over 6000 episodes and the federated method was experimented with the aggregation period of FL of 1, 10, and 100. Comparing the results of the DQN and DDPG methods, it is evident that DDPG convergence is faster and smoother than that of DQN, further providing more consistent performance on the vehicle. It can also be confirmed that the DDPG method can explore more efficient action spaces in continuous problems because the DQN method deals only with discrete action spaces.

In the single-agent method, all vehicles learn the global model by transmitting the observed state to the VECS controller, with a large communication overhead. We can see that the FL and single-agent methods have similar performance. In the FL method, the aggregation period is more frequent and the convergence speed is faster, but the communication overhead increases. However, the FL method (when the aggregation period is 100) has up to 0.3 times less communication overhead between vehicles and the VECS controller than single-agent methods. Moreover, we can observe that the FL method significantly improves the performance by approximately 35% compared to the multi-agent method. The FL and multi-agent methods differ by 0.09 times in communication overhead. This is because the multi-agent method trains only its model based on the observed state of each vehicle and does not share model weights with other vehicles. 

**Figure 4:**
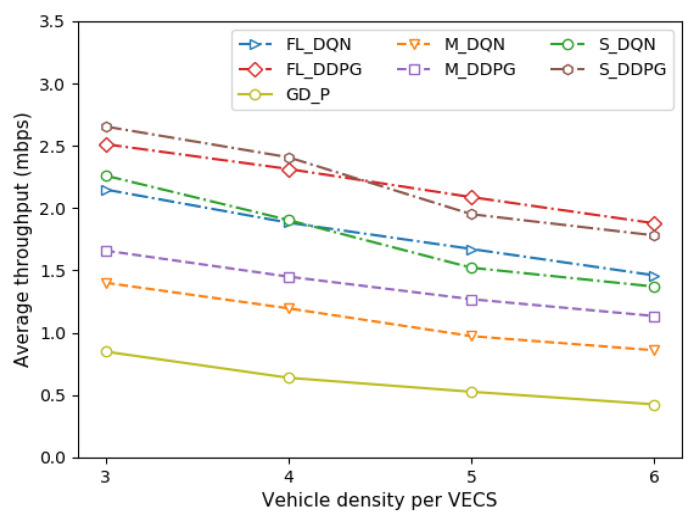
Comparison of average throughput under different vehicle densities.

**Figure 5:**
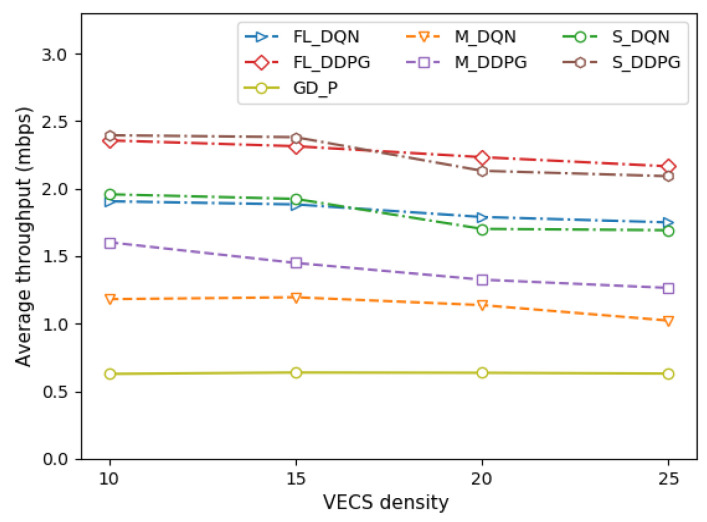
Comparison of average throughput under different VECS densities.

Figure 4 depicts the average throughput of the vehicle under different vehicle densities per VECS. Figure 5 depicts the average throughput of the vehicle under different VECS densities in the VEC system. Throughput is defined as the total amount of tasks processed by offloading (Equation (1)). It can be observed that the average throughput decreases as the vehicle and VECS densities increase, and all methods tend to be similar. As the vehicle density increases, the intra-cell interference increases (Equation (2)). Similarly, as the VECS density increases, the inter-cell interference increases (Equation (2)). Therefore, an increase in the overall channel interference decreases the average throughput of the vehicle, thereby resulting in a decrease in the system throughput. 

The single-agent, multi-agent, and federated versions of the DDPG method are approximately 19–20% better than those of the DQN method. Although the FL method performs as well as the single-agent method, the FL method is preferred when the vehicle and the VECS are dense. This means that it is difficult to make an optimal decision as the number of VEC systems increases. Comparisons with the GD-P method show that regardless of the amount of transmission power allocated by the vehicle, its performance is poor in terms of throughput owing to channel interference.

**Figure 6:**
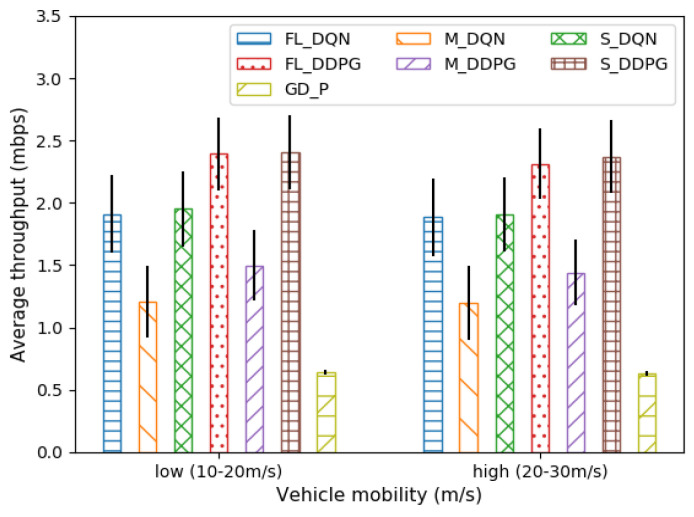
Comparison of average throughput under vehicle mobility.

Figure 6 depicts the average throughput when the vehicle is low and high in mobility. The mobility of the vehicle is related to the Doppler frequency (Equation (6)), which is a variable related to small-scale fading. The higher the mobility of the vehicle, the higher the Doppler frequency. As a result, the temporal correlation of the channel may decrease (Equation (4)), and fast fading may cause performance degradation. However, we can observe that the average throughput of all methods gradually decreases to approximately 1–4%. Therefore, we can see that these offloading methods are robust against vehicle mobility. 

**Figure 7:**
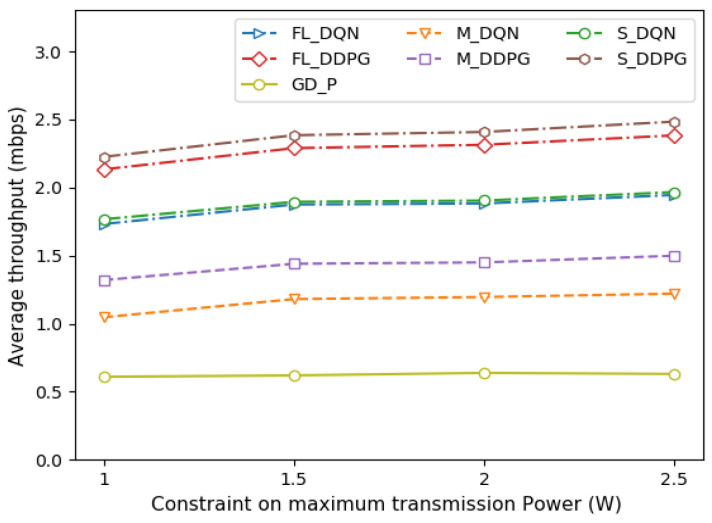
Comparison of average throughput under different power constraints.

Figure 7 depicts the average throughput of the vehicle under various constraints on maximum transmission power. As the maximum transmission power of the vehicle increases, the number of tasks that can be offloaded increases. Therefore, it can be seen that as the transmission power increases, throughput increases. However, the throughput does not increase as much as the transmission power increases. This is because channel interference increases as the transmission power increases. The allocation of transmission power of a vehicle considering channel interference has a more important effect on performance than the constraint on the maximum transmission power of the vehicle.

**Figure 8:**
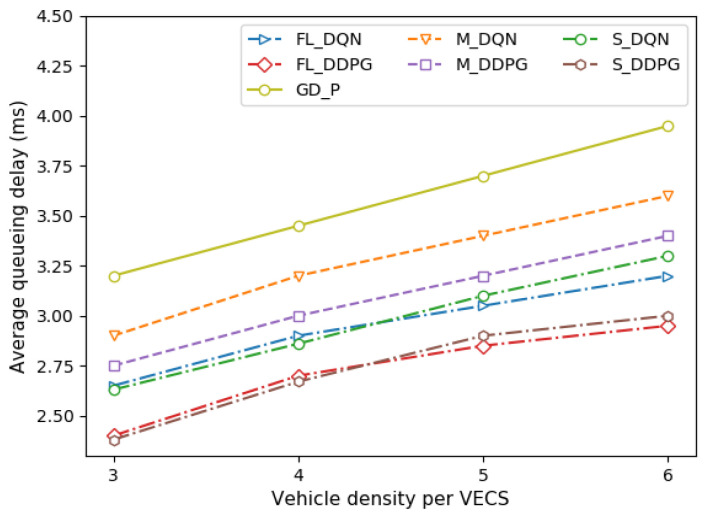
Comparison of queueing delay under different vehicle densities.

Figure 8 depicts the queueing delay of the vehicle under different vehicle densities per VECS. The queueing delay is affected by the vehicle throughput. High throughput means that there is little work to be processed in the queue. Therefore, this can reduce the queueing delay by reducing the waiting time in the queue, which results in QoS improvement. As illustrated in Figure 4, the queueing delay is low in the order of high throughput, and as the vehicle density increases, the throughput decreases; thus, the queueing delay also increases. 

**Figure 9:**
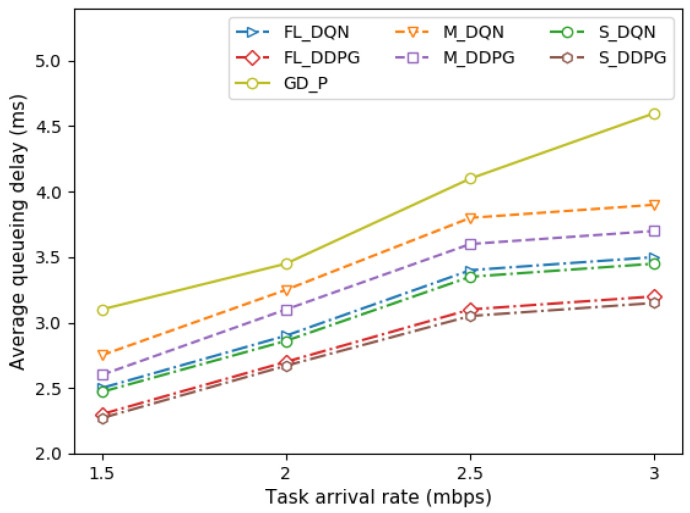
Comparison of queueing delay under different task arrival rates.

In addition, Figure 9 depicts the queueing delay of the vehicle under different task arrival rates. Even if the load of the vehicle increases as the task arrival rate increases, the queueing delay increases slightly. This is because we allocated the transmission power for offloading by considering the queue state of the vehicle, that is, the stochastic task arrival rate.

## 6. Conclusions

In this study, we proposed offloading with a power control method to maximize the system throughput under the power constraints of a vehicle by dynamically allocating transmission power for offloading. We leveraged the FL based on the DDPG framework. 

From the experiments, we found that channel interference owing to the density of the vehicle and VECS significantly influence the throughput. Increasing overall channel interferences due to increased densities of vehicles and VECSs decrease the throughput and increase queueing delay of the vehicle. However, the mobility of the vehicle is robust in terms of throughput. In addition, comparing the single-agent, multi-agent, and FL versions of the DRL methods, the FL-based method shows the best performance in terms of throughput and queueing delay. The FL-based method performs as well as the single-agent method, but the FL method is preferred when the vehicle and VECS are dense. This is because it is difficult to make an optimal decision as the number of VEC systems increases. The DDPG methods are superior to the DQN methods in terms of convergence and performance, especially in the continuous space. Through comparison with the greedy method, it was confirmed that allocating transmission power of the vehicle in consideration of channel interference affects performance. Therefore, it is proved that the proposed method, FL-DDPG, shows the best performance compared with other methods.

The FL-based DDPG method can be applied to several problems such as channel management and task scheduling in VEC systems. In future research, we will extend the research offloading method, considering V2I (vehicle-to-infrastructure) and V2V (vehicle-to-vehicle) links to manage a vehicle’s channel. In addition, we plan to predict the environment or the state of the vehicle to increase the performance in terms of power management [26,27].

## Figures and Tables

**Table 1:** Simulation parameters.

Parameter	Value
Number of VECSs	15
Density of vehicles per VECS	4
Mobility of vehicles ($v_{n}$)	20 m/s
Channel bandwidth (B)	1 MHZ
Background noise power $\left(\sigma^{2}\right)$	$10^{-9}\;W$
Maximum transmission power of vehicle $n\;\left(P_{max}\right)$	2 W
Task arrival rate $\left(\lambda_{n}\right)$	2 Mbps
Length of time slot$\;\left(\tau_{0}\right)$	1 ms
Path loss model	$128.1+37.6\;\log_{10}\left(dist\right)$
Discount factor of long-term reward (γ)	0.99
Update parameter for target network (τ)	0.001

## Data Availability

Not applicable.
